# High-Dose Acetaminophen Alters the Integrity of the Blood–Brain Barrier and Leads to Increased CNS Uptake of Codeine in Rats

**DOI:** 10.3390/pharmaceutics14050949

**Published:** 2022-04-27

**Authors:** Junzhi Yang, Robert D. Betterton, Erica I. Williams, Joshua A. Stanton, Elizabeth S. Reddell, Chidinma E. Ogbonnaya, Emma Dorn, Thomas P. Davis, Jeffrey J. Lochhead, Patrick T. Ronaldson

**Affiliations:** 1 Department of Pharmacology and Toxicology, College of Pharmacy, University of Arizona, Tucson, AZ 85721, USA; jzyang345@email.arizona.edu (J.Y.); davistp@email.arizona.edu (T.P.D.); 2 Department of Pharmacology, College of Medicine, University of Arizona, Tucson, AZ 85724, USA; rdbetter@email.arizona.edu (R.D.B.); eiwilliams@email.arizona.edu (E.I.W.); joshuastanton@email.arizona.edu (J.A.S.); ekurilko@email.arizona.edu (E.S.R.); ceogbonn@email.arizona.edu (C.E.O.); emmadorn@email.arizona.edu (E.D.)

**Keywords:** acetaminophen, blood–brain barrier, claudin-5, CNS drug delivery, opioids, tight junction

## Abstract

The consumption of acetaminophen (APAP) can induce neurological changes in human subjects; however, effects of APAP on blood–brain barrier (BBB) integrity are unknown. BBB changes by APAP can have profound consequences for brain delivery of co-administered drugs. To study APAP effects, female Sprague–Dawley rats (12–16 weeks old) were administered vehicle (i.e., 100% dimethyl sulfoxide (DMSO), intraperitoneally (i.p.)) or APAP (80 mg/kg or 500 mg/kg in DMSO, i.p.; equivalent to a 900 mg or 5600 mg daily dose for a 70 kg human subject). BBB permeability was measured via in situ brain perfusion using [^14^C]sucrose and [^3^H]codeine, an opioid analgesic drug that is co-administered with APAP (i.e., Tylenol #3). Localization and protein expression of tight junction proteins (i.e., claudin-5, occludin, ZO-1) were studied in rat brain microvessels using Western blot analysis and confocal microscopy, respectively. Paracellular [^14^C]sucrose “leak” and brain [^3^H]codeine accumulation were significantly enhanced in rats treated with 500 mg/kg APAP only. Additionally, claudin-5 localization and protein expression were altered in brain microvessels isolated from rats administered 500 mg/kg APAP. Our novel and translational data show that BBB integrity is altered following a single high APAP dose, results that are relevant to patients abusing or misusing APAP and/or APAP/opioid combination products.

## 1. Introduction

The blood–brain barrier (BBB) is essential for maintenance and regulation of brain homeostasis. The BBB selectively and dynamically regulates solute exchange between the central nervous system (CNS) and systemic circulation and simultaneously restricts entry of harmful substances into the brain. The BBB is susceptible to disruption or modulation by stress factors including disease states (e.g., ischemic stroke [1,2,3], inflammatory pain [4,5,6], and Alzheimer’s disease [7,8,9,10]), as well as the presence of circulating intrinsic regulators (e.g., hormones [11,12]) and xenobiotics including environmental toxins [13,14,15] and drugs [16,17]). For example, morphine is an inducer of ATP-binding cassette transporters at the BBB including P-glycoprotein and breast cancer resistance protein [18,19,20], which reduces blood-to-brain permeability of many drugs and is suggested to be an underlying mechanism for development of opioid tolerance following chronic use. Besides opioid analgesics, other common psychoactive substances including cocaine [21,22], nicotine [23,24], alcohol [25,26], methamphetamine [27,28,29,30] and methylenedioxymethamphetamine (MDMA, “Ecstasy”) [31,32] have been shown to modulate or damage the BBB in varying capacities by changing expression levels and/or post-translational modification status of endothelial transporters or tight junction proteins, or by disruption of functioning tight junction BBB protein assemblies. Indeed, modifications to BBB function by drugs of abuse and polypharmacy can have serious consequences leading to dysregulation of brain homeostasis [33,34], neuronal degeneration [9,10], and concomitant drug–drug interactions [35,36,37]. In contrast, investigations on the potential of therapeutics that are not classified as drugs of abuse to alter the BBB is lacking.

Acetaminophen (i.e., paracetamol, APAP) is a potent antipyretic and analgesic agent and one of the most commonly used and abused medications, both in the United States and worldwide. According to the Consumer Healthcare Products Association (CHPA), 23% of adults in the United States use an APAP-containing medication on a weekly basis [38]. Although APAP is generally regarded as safe when taken as directed, it is often consumed in excessive amounts. A five-year national survey over the period of 2011–2016 indicated an overall 6.3% rate of overuse of APAP-containing products among participants that significantly exceeded the 4000 mg recommended daily dose [39]. In line with this survey, Blieden and colleagues reported that approximately 6% of their recruited chronic pain patients each year in the United States were prescribed by their physician with an over-4000 mg daily dosage of APAP [40]. Similarly, an Australian study, which recruited chronic non-cancer pain patients, described that 6.1% of participants used considerably more than 4000 mg of APAP per day during the week-long study, and 8.0% of participants consumed quantities of APAP higher than 4000 mg, up to 9540 mg, in a given day [41].

According to StatPearls in 2021, approximately 50% of all APAP-related hospital visits were related to unintentional overdose [42]. Indeed, overuse of APAP is strongly associated with the use of concomitant medications [43]. This is not surprising given that a substantial portion of the over-six-hundred APAP-containing medications are combination drugs, a classic example of polypharmacy. Notably, opioids including hydrocodone (i.e., Vicodin^®^), oxycodone (i.e., Percocet^®^), and codeine (i.e., Tylenol #3^®^) are commonly co-administered with APAP. A cohort study in 1998–2003 in the United States found that opioid-containing products were involved in 63% of unintentional APAP overdoses [44]. In fact, the hydrocodone–APAP combination was the most frequently prescribed medication between the years of 2006–2011 [45], and a medical surveillance in 2004–2005 study identified hydrocodone–APAP and oxycodone–APAP combination medications as some of the most commonly implicated drugs in medication-related adverse events for emergency visits [46]. Both hydrocodone–APAP and oxycodone–APAP combinations are now classified as Schedule II controlled substances by the United States Drug Enforcement Administration (DEA), leaving the codeine–APAP combination as the only Schedule III opioid-containing analgesics on market [47]. The Schedule III classification allows for less stringent prescription regulations (i.e., written, verbal, or electronic prescriptions), and is currently a cause for concern in pediatric medicine [48]. According to analysis of death-certificate data of 2010, opioids were involved in 75.2% of all pharmaceutical overdose deaths of the year, and high-dose APAP was implicated in 2.4% of deaths due to opioid overdose [49]. It is important to note that complete toxicology analyses were not performed in all patients in this study, suggesting that high APAP levels may be involved in a greater percentage of patient deaths.

Considering the prevalence of APAP use and the fact that opioids are frequently co-administered with APAP, it is of critical importance to understand its effects on the BBB. Hepatoxicity of APAP has been extensively studied, and the consumption of APAP has been linked to neurological alterations without achieving acute liver injury [50,51]; however, there is a marked gap in knowledge regarding effects of APAP directly at the BBB. Furthermore, the current opioid epidemic indicates an urgent need to expand our knowledge on effects of APAP at the BBB in order to understand how it impacts the delivery of concomitantly administered drugs (i.e., opioids) into the CNS. In this study, we hypothesized that APAP modulates paracellular permeability of the BBB by disrupting tight junction barrier proteins at the brain microvascular endothelium, thereby exacerbating CNS opioid exposure through the “leak” of codeine into the brain. To test this hypothesis, we studied, in vivo, paracellular solute “leak” and localization/expression of critical tight junction proteins at the BBB (i.e., claudin-5, occludin and ZO-1) following APAP administration. Our work demonstrated, for the first time, that an acute high dosage (500 mg/kg) of APAP increases paracellular leak of the BBB to both sucrose (i.e., a vascular marker) and codeine, a commonly prescribed opioid that is concomitantly administered with APAP. Of particular significance, low-dose APAP (80 mg/kg) did not have significant effects on BBB permeability to sucrose or codeine. In addition to BBB permeability changes in response to high-dose APAP, we showed that tight junction protein complex disruption by acute APAP exposure is characterized by altered expression of claudin-5 in rat brain microvessels.

## 2. Materials and Methods

### 2.1. Animals and Treatments

Animal protocols were approved by the University of Arizona Institutional Animal Care and Use Committee (Protocol #18-377; Approval Date: 25 February 2021) and were conducted in compliance with both National Institutes of Health and Animal Research: Reporting In Vivo Experiments (ARRIVE) guidelines. Female Sprague–Dawley rats were purchased from Envigo (Madison, WI, USA). At the time of experimentation, rats were 3–4 months old with body weights of 200–250 g. We purposely focused our study on female experimental animals to enable robust comparison with our previous study on APAP effects at the BBB [52]. Animals were housed under controlled conditions (22.2–22.4 °C; 50% relative humidity; 12 h light/dark cycle) with free access to food and water for a minimum of seven days. For low- and high-dose drug treatments groups, APAP (Millipore-Sigma, St. Louis, MO, USA) was dissolved in vehicle (100% dimethyl sulfoxide; DMSO) to achieve doses of 80 mg/mL and 500 mg/mL, respectively. Animals received a single intraperitoneal (i.p.) injection of either APAP or vehicle (1 mL/kg). Three hours after treatment and prior to further experimentation, animals were anesthetized with 100 mg/mL ketamine with 10 mg/mL xylazine (i.p.).

### 2.2. In Situ Brain Perfusion

In situ brain perfusion was performed as described previously by our laboratory [53,54]. Briefly, after anesthesia, experimental animals (*n* = 6) were given an i.p. dose of heparin (10,000 U/kg) to prevent coagulation. Common carotid arteries were exposed and bilaterally canulated to connect to the perfusion circuit. Both jugular veins were severed to provide drainage. The perfusion buffer (117 mM NaCl, 4.7 mM KCl, 0.8 mM MgSO_4_, 1.2 mM KH_2_PO_4_, 2.5 mM CaCl_2_, 10 mM d-glucose, 3.9% dextran (75,000 g/mol), and 1.0 g/L bovine serum albumin; pH 7.4) was warmed to 37 °C and oxygenated with 95% O_2_ and 5% CO_2_. Evan’s blue dye was added to the perfusion buffer as a visual indicator for tight junction intactness. Rat brains were perfused for 10 min at a total flow rate of 3.6 mL per min under a perfusion pressure of 95 to 105 mmHg. In control experiments, we monitored EKG and respiratory waveforms in animals subjected to in situ brain perfusion for up to 30 min. In all animals, these physiological parameters remained within normal limits, which implies that our in situ brain perfusion method allows for evaluation of BBB integrity and permeability in a stable, well-controlled environment for the entire duration of the perfusion. For measurement of the BBB paracellular leak, [^14^C]sucrose (Specific Activity = 0.5000 mCi/mL; PerkinElmer Life and Analytical Sciences, Boston, MA, USA) was used as a vascular marker and infused into the perfusate at 0.5 mL per min using a slow-drive syringe pump (Harvard Apparatus, Cambridge, MA, USA). For the measurement of cerebral exposure to opioids, [^3^H]codeine (Specific Activity = 2.0000 mCi/mL; Research Triangle Institute, Research Triangle, NC, USA) was infused into the perfusion circuit with identical settings to the sucrose experiments. Immediately after perfusion, rat brains were extracted and processed by removing cerebellum, meninges and choroid plexus. The processed brains were divided into three parts and solubilized for two days using 1 mL TS2 tissue solubilizer. At this time, a 2 mL Optiphase SuperMix liquid scintillation cocktail (PerkinElmer Life and Analytical Sciences) was added to each tube to enable the measurement of radioactivity and 100 μL 30% (*v*/*v*) glacial acetic acid was added to quench background counts. Radioactivity was measured with a 1450 Liquid Scintillation and Luminescence Counter (PerkinElmer Life and Analytical Sciences) and reported as brain-to-perfusate radioactivity ratios (Rbr %; pmol/mg brain tissue) by dividing the measured amount of radioisotope in brain per brain weight by the known amount of radioisotope in the perfusate:

RBr (%; pmol/mg brain tissue) = C_Brain_/C_Perfusate_ × 100% (1)

The brain vascular volume in rats has been previously shown to range between 6 and 9 μL/g of brain tissue in perfusion studies utilizing a saline-based bicarbonate buffer [55]. Since brain tissue was processed immediately after perfusion with radiolabeled substrate, all uptake values obtained for [^14^C]sucrose or [^3^H]codeine required correction for brain vascular volume (i.e., 8.0 μL/g brain tissue as calculated from data reported by Takasato and colleagues [55]).

### 2.3. Microvessel Isolation

Rat brain microvessels were isolated according to a protocol developed and published by our laboratory [56]. Briefly, animals (*n* = 9) were euthanized with ketamine/xylazine and decapitated. Brains were removed, processed (i.e., removal of cerebellum, meninges and choroid plexus), and homogenized in ice-cold brain microvessel buffer (pH 7.4) with 0.1% protease inhibitor cocktail (Millipore-Sigma, St. Louis, MO, USA). After thoroughly mixing the brain homogenate with 26% dextran (75,000 g/mol), samples were centrifuged at 6500× *g* at 4 °C for 30 min. Following centrifugation, the supernatant was aspirated, and pellets were resuspended in the same buffer, mixed with 26% dextran, and were once again centrifuged for 30 min under the same conditions. The microvessel-enriched pellets from the second centrifugation were resuspended in Pierce™ IP Lysis Buffer (Thermo Fisher Scientific, Waltham, MA, USA) with cOmplete™ Mini Protease Inhibitor Cocktail (Roche, Basel, Switzerland), and Phosphatase Inhibitor Cocktail II and III (Research Products International) and stored at −80 °C until further analysis.

### 2.4. Western Blotting

The total protein concentration of the samples was measured with the Pierce™ BCA Protein Assay Kit (Thermo Fisher Scientific, Waltham, MA, USA). Western blots were performed using the Criterion™ XT Bis-Tris electrophoresis and blotting system (Bio-Rad Laboratories, Hercules, CA, USA). After electrophoresis and protein transfer, the blotting membranes were blocked with SuperBlock™ Blocking Buffer (ThermoFisher Scientific, Waltham, MA, USA) and incubated with appropriate primary antibodies overnight at 4 °C. Primary antibodies that were used in these experiments were designed to detect claudin-5 (4C3C2; Cat #35-2500; 0.5 mg/mL; 1:2000 dilution), occludin (Cat #40-6100; 0.25 mg/mL; 1:500 dilution), and ZO-1 (Cat #40-2200; 0.25 mg/mL; 1:1000 dilution) and were purchased from Thermo Fisher Scientific (Waltham, MA, USA). As a loading control, α-tubulin was detected using a commercially available primary antibody (DM1A; Cat #ab7291; 1.0 mg/ml; 1:1000 dilution) from Abcam, Inc. (Cambridge, MA, USA). After overnight incubation, membranes were washed and incubated with horseradish peroxidase (HRP)-conjugated AffiniPure Goat Anti-Rabbit secondary IgG antibody (Cat #111-035-144; 1:5000 dilution) or HRP-conjugated AffiniPure Goat Anti-Mouse secondary IgG antibody (Cat #115-035-166; 1:5000 dilution) from Jackson ImmunoResearch Laboratories. Inc. (West Grove, PA, USA). The specificity of claudin-5, ZO-1, and occludin primary antibodies has been confirmed for Western blotting experiments by demonstrating the enrichment of specific protein bands corresponding to each tight junction protein in rat brain microvessel samples as compared to rat skeletal muscle homogenate (Figure S1A). Protein signals were detected with SuperSignal™ West Pico PLUS Chemiluminescent Substrate (Thermo Fisher Scientific, Waltham, MA, USA) and imaged using the ChemiDoc™ Touch Imaging System from Bio-Rad Laboratories. Densitometry analysis of protein bands was performed using ImageJ (Wayne Rasband, Research Services Branch, National Institute of Mental Health, Bethesda, MD, USA). Band intensities of tight junction proteins (i.e., claudin-5, occludin, ZO-1) were normalized to those of the loading control (i.e., α-tubulin) for statistical analysis.

### 2.5. Immunofluorescence Staining

Three hours after treatment with vehicle or APAP, rats were anesthetized with ketamine/xylazine and decapitated. The brain was immediately removed and snap-frozen at −75 °C in isopentane on dry ice. Cryosections (20 μm) were mounted onto glass slides and stored at −80 °C until needed for staining. Sections were fixed in methanol at −20 °C, blocked in PBS with 0.3% Triton X-100 + 5% goat serum and then incubated in primary antibody overnight at 4 °C. Primary antibodies used for immunofluorescence were rabbit anti-ZO-1 (Cat #40-2200; 1:750 dilution; Thermo Fisher Scientific, Waltham, MA, USA), mouse anti-claudin-5 (Cat #35-2500; 1:500 dilution; Thermo Fisher Scientific, Waltham, MA, USA), and mouse anti-occludin (OC-3F10; Cat #33-1500; 0.5 mg/mL; 1:500 dilution; Thermo Fisher Scientific, Waltham, MA, USA). These antibodies were detected with Alexa 488 goat anti-rabbit (Cat #A11008; 1:500 dilution; Thermo Fisher Scientific, Waltham, MA, USA) and Alexa 568 goat anti-mouse (Cat #A11004; 1:500 dilution; Thermo Fisher Scientific, Waltham, MA, USA). DyLight 649-tomato lectin (Cat #DL-1178-1; Vector Laboratories, Burlingame, CA, USA) was used to label and visualize the cerebral vasculature. Control experiments were conducted by incubating sections in the presence of secondary antibody only (i.e., no primary antibody controls). Data for these control experiments are presented in Figure S1B.

### 2.6. Confocal Microscopy

Confocal microscopy was performed on a Leica SP8 confocal microscope (Leica Biosystems, Wetzlar, Germany) with 488 and 552 nm excitation lasers. Emitted light was detected with a Leica hybrid detector (HyD). Images were acquired from randomly chosen areas of the somatosensory cortex between 2 mm rostral to 1 mm caudal relative to Bregma. Images from control and treated animals were acquired using matching laser power and gain settings with emission windows set to prevent bleed-through between fluorophores. Adjustments for brightness and contrast levels were performed with ImageJ software (NIH) in an identical manner for both control and treated images.

### 2.7. Statistical Analysis

In situ brain perfusion brain-to-perfusate radioactivity ratios (RBr %) and Western blot densitometric analysis data (normalized to loading control) were reported as mean ± S.D. Statistically significant differences between control and treatment groups were determined with one-way analysis of variance (ANOVA) followed by post hoc two-tailed unpaired homoscedastic Student’s *t*-test to examine differences between groups. A value of *p* < 0.05 was considered to be statistically significant.

## 3. Results

### 3.1. Increased BBB Paracellular “Leak” in Response to High-Dose APAP Treatment

**Figure 1:**
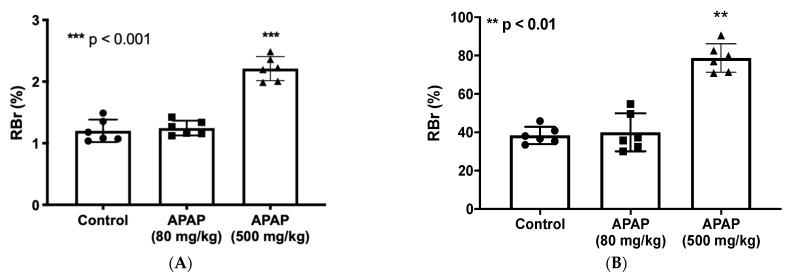
BBB paracellular permeability to sucrose and codeine is increased at three hours following high-dose APAP administration. (**A**): In situ brain perfusion with [^14^C]sucrose as a vascular paracellular permeability marker shows significantly elevated radioactivity level represented in brain-to-perfusate radioactivity ratios (RBr %) in brains of high-dose APAP-treated rats (*n* = 6) in comparison to vehicle or low-dose APAP-treated rats. No significant change radioactivity level was measured in the low-dose APAP (80 mg/kg) treated rats. (**B**): In situ perfusion with [^3^H]codeine following APAP injection shows significantly higher radioactivity represented in brain-to-perfusate radioactivity ratios (RBr %) in brains of APAP (500 mg/kg)-treated rats (*n* = 6) in comparison to vehicle. Data are expressed as mean ± S.D. (** *p* < 0.01; *** *p* < 0.001).

To investigate whether there is a change in BBB paracellular permeability (i.e., “leak”) in the presence of APAP, radiolabeled sucrose (i.e., [^14^C]sucrose) was used. We administered 80 mg/kg of APAP, a low dose emulating approximately 900 mg for an average-weight human adult (i.e., 70 kg), and 500 mg/kg of APAP, an acute dosage equivalent to approximately 5600 mg for an average-weight human adult (i.e., 70 kg), for the low- and high-dose treatments, respectively, 3 h prior to in situ brain perfusion with [^14^C]sucrose. As shown in Figure 1A, only the high-dose APAP treatment resulted in significantly elevated codeine and sucrose radioactivity in perfused rat brains, which indicates disruption of BBB functional integrity leading to paracellular leak.

### 3.2. Elevated CNS Exposure to Codeine after APAP Treatment

To investigate whether the increase in BBB paracellular permeability due to APAP stress contributes to elevated CNS exposure to drugs, we utilized in situ brain perfusion to measure [^3^H]codeine uptake into brain tissue 3 h following APAP injection (80 mg/kg or 500 mg/kg; i.p.). Codeine was purposely selected for these experiments since it is commonly co-administered with APAP in combination products including, but not limited to, Tylenol #3^®^. It is also an opioid analgesic drug that accesses the CNS primarily by passive diffusion and not by facilitated transport processes [54,57], which renders it an ideal drug to use in the evaluation of altered BBB functional integrity by APAP. Codeine radioactivity levels measured in brain tissue of high-dose APAP (500 mg/kg)-treated rats were significantly increased as compared to those of low-dose APAP (80 mg/kg) or vehicle controls (Figure 1B). This indicates that concomitantly administered medications such as high-dose APAP and codeine can interact at the BBB, thereby causing a serious risk of adverse effects associated with the codeine due to the elevated brain penetration of this opioid analgesic drug in the presence of a high dose of APAP.

### 3.3. Increased Claudin-5 Expression in Brain Microvessel after High-Dose APAP Treatment

**Figure 2:**
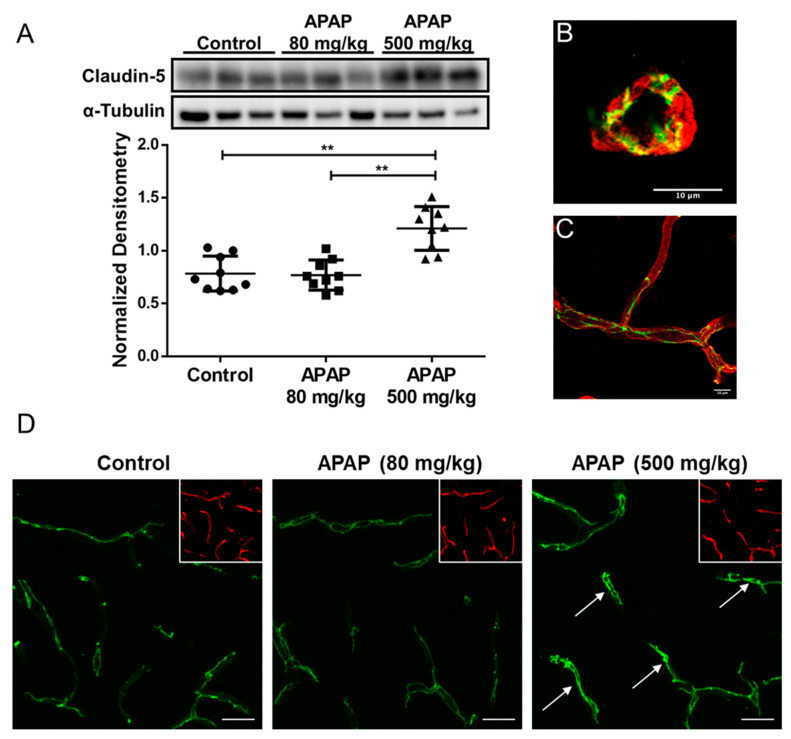
Claudin-5 level increases in BBB microvessel endothelial cells three hours after high-dose APAP treatment. (**A**) Western blot (**top**) and densitometric analysis (**bottom**) of claudin-5 levels in isolated brain microvessel of rats (*n* = 9) treated with low-dose APAP (80 mg/kg) and high-dose APAP (500 mg/kg) shows statistical significance between the three groups (*p* = 0.000012), as well as between vehicle and the high-dose groups (*p* = 0.00018; Student’s *t*-test) and between low-dose and high-dose groups (*p* = 0.000074). No statistical significance was detected between the vehicle and the low-dose APAP group (*p* = 0.84). Densitometric data are expressed as mean ± S.D. (** *p* < 0.0001). High magnification confocal microscopy images of a cross-section (**B**) and a longitudinal section (**C**) of a cortical microvessel showing claudin-5 localization (green) within the microvessel labeled with the vascular marker tomato lectin (red). (**D**) confocal microscopy images indicate higher overall claudin-5 expression in cortical microvessel of rats treated with high-dose APAP (500 mg/kg) than vehicle, but not in those treated with low-dose APAP (80 mg/kg). Arrows show sites of claudin-5 upregulation. Microvessels were stained with lectin (red) and are shown in the inset to demonstrate vascular localization of claudin-5. Scale bar = 20 μm.

Since claudin-5 is considered to be the primary determinant of BBB tight junction integrity [17], we measured changes in claudin-5 content in rat brain microvessels 3 h after an acute low dose (80 mg/kg; i.p.) or high dose (500 mg/kg; i.p.) of APAP in accordance with our in situ brain perfusion experiment. Western blot analysis of microvessel samples indicated a significant elevation in claudin-5 expression in response to high-dose APAP treatment as compared to low-dose APAP (*p* < 0.0001) or vehicle-treated rats (*p* < 0.0001) (Figure 2A). Both a cross-section (Figure 2B) and longitudinal section (Figure 2C) of a rat brain microvessel show localization of claudin-5 at endothelial cell margins, evidence for its role as a critical constituent of tight junction protein complexes. Confocal imaging of microvessels in rat brain cryosections corroborated with our Western blot data, demonstrating an overall increase in claudin-5 staining throughout cortices of rats three hours after high-dose APAP treatment as compared to vehicle (Figure 2D).

**Figure 3:**
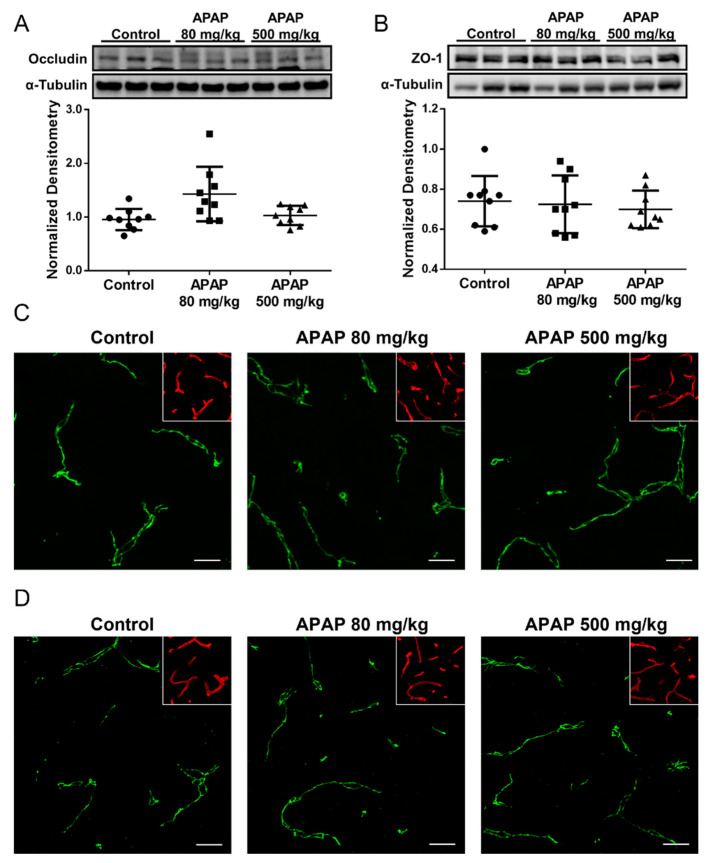
Occludin and ZO-1 levels remain unchanged three hours following APAP treatment. (**A**) Western blot (**top**) and densitometric analysis (**bottom**) shows comparable occludin levels in isolated brain microvessel of APAP (500 mg/mL)-treated and control rats (*n* = 9). Densitometric data are expressed as mean ± S.D. (**B**) Western blot (**top**) and densitometric analysis (**bottom**) shows comparable ZO-1 levels in isolated brain microvessel of APAP (500 mg/mL)-treated and control rats (*n* = 9). Densitometric data are expressed as mean ± S.D. (**C**) Confocal microscopy shows similar overall occludin expression in cortical microvessel of rats treated with APAP (80 or 500 mg/kg) as vehicle. Microvessels were stained with lectin (red) and are shown in the inset to demonstrate vascular localization of occludin. Scale bar = 20 μm. (**D**) Confocal microscopy shows similar overall ZO-1 expression in cortical microvessel of rats treated with APAP (80 or 500 mg/kg) as vehicle. Microvessels were stained with lectin (red) and are shown in the inset to demonstrate vascular localization of ZO-1. Scale bar = 20 μm.

We also evaluated APAP effects on other tight junction proteins including the transmembrane protein occludin and the intracellular accessory protein zonula occludens-1 (ZO-1). In contrast to our results with claudin-5, Western blot analysis and confocal microscopy of occludin (Figure 3A,C) and ZO-1 (Figure 3B,D) in cortical microvessels showed no detectable change in either APAP-treated or untreated rats at the three-hour timepoint. Taken together, these observations suggest that the changes in claudin-5 is likely involved in the process leading up to the observed BBB paracellular “leak” in response to high-dose APAP treatment, while occludin and ZO-1 are likely not responsible for the disruption of BBB tight junctions in the acute phase of the high-dose APAP stress response.

## 4. Discussion

The BBB plays an essential role in regulation of brain homeostasis and preservation of the optimal microenvironment for proper neuronal function. Dysregulation of BBB permeability is associated with many pathologies [17] and can lead to increased vulnerability (i.e., leak) of the brain to harmful substances in the systemic circulation [52,54,57,58,59,60,61,62]. For example, peripheral inflammatory pain can cause BBB dysfunction that is manifested by increased paracellular “leak” to circulating small molecule solutes including drugs [54]. Such enhancement in paracellular permeability was shown to result from modulation of tight junction protein complexes at the brain microvascular endothelium. Claudins, as well as occludin, are critical transmembrane tight junction proteins at the BBB that form protein complexes responsible for physically sealing paracellular gaps between adjacent endothelial cells [17,63]. Changes to protein expression levels of claudins and occludin are frequently observed in modulation of stress responses by tight junctions at the BBB [17]. ZO-1 is an important intracellular accessory protein at the tight junction that links transmembrane proteins to the actin cytoskeleton, thus forming complex networks essential for the dynamic regulation of the BBB [17]. In the present study, we are the first to demonstrate that acute treatment of APAP disrupts tight junction protein complexes in the cerebral microvasculature, as evidenced by an increase in paracellular permeability at the BBB and an upregulation of claudin-5. We believe these changes are due specifically to APAP administration rather than the DMSO solvent. Our previous work has shown that BBB permeability to sucrose or small molecule drugs in control (i.e., untreated animals) is not altered in vivo following acute (i.e., up to 3 h) administration of DMSO vehicle [53,64]. Preliminary time-point experiments showed that this increase in claudin-5 levels was sustained until 6 h after APAP treatment and returned to baseline by 12 h (data not shown). Additionally, as increased levels of claudin-5 in the systemic circulation have been implicated in BBB breakdown [65,66], we conducted an ELISA analysis of rat plasma that detected a slight but statistically significant (*p* = 0.03) increase in plasma claudin-5 protein levels at 3 h following APAP administration (data not shown). In contrast, occludin and ZO-1 were not affected by APAP treatment, indicating that claudin-5 is likely the primary tight junction protein that controls the response to high-dose APAP-induced stress and, by extension, enhanced paracellular “leak” associated with APAP administration.

Although a simultaneous increase in both claudin-5 expression and BBB permeability may seem paradoxical, it is essential to note that many publications in the scientific literature have reported that claudin-5 upregulation is associated with an increase in barrier permeability or neuronal injury due to pathophysiological stressors. For example, studies in the kainic acid model of temporal lobe epilepsy demonstrated a significant increase in claudin-5 levels 24–72 h after kainic acid injection in correspondence with neuronal injury [67]. Our laboratory’s studies in a rat model of peripheral inflammatory pain showed a 6-fold increase in microvessel claudin-5 levels with BBB paracellular “leak” but no detectable change in claudin-5 localization [6]. Further investigation into the underlying mechanism revealed that transforming growth factor (TGF)-β-signaling mediated this disruption of the BBB that was manifested by sucrose extravasation and claudin-5 upregulation at the brain microvascular endothelium [53]. Claudin-5 is also elevated in epithelial tight junctions in lung tissue isolated from alcoholic rats with diminished barrier functions, likely a result of disrupted protein-protein interactions between claudins [68]. Interestingly, overexpression of claudins 1 and 3 in stably transfected NIH/3T3 or IB3.1 cells leads to a decrease in permeability to dextrans but overexpression of claudin-5 in these same cells results in an increase in permeability to dextrans [69]. Additionally, it is essential to consider that changes in monomeric tight junction protein expression following exposure to a pathological or pharmacological stressor may reflect altered tight junction integrity via collapse of oligomeric structures. Indeed, claudin-5 has been shown to form oligomers in HEK293 cells transfected with native claudin-5 [70] or chimeric claudin-5 proteins [71]. The ability of claudin-5 to assemble into higher order structures was proposed to be required for the formation of tight junction strands and, subsequently, the sealing function of tight junction protein complexes in endothelial cells [70,71,72]. These findings highlight the fact that elevated claudin-5 protein expression in brain microvascular endothelial cells is not necessarily indicative of improved neurovascular integrity. Rather, any change in claudin-5 expression, regardless of directionality, can be reflective of tight junction dysregulation and paracellular leak, depending on the specific pathological or mechanistic processes involved and the resulted state of functional intact nature of tight junction assembly. Future work by our group will elucidate the mechanism of action pertaining to the upregulation of claudin-5 in response to APAP treatment.

APAP may also affect BBB permeability through interactions with phospholipids in the plasma membrane. APAP directly binds to phospholipids and significantly increases membrane fluidity in a dose-dependent manner [73]. Alterations in physicochemical properties of the plasma membrane are likely to alter the function of the tight junction protein complex by dynamically affecting the organization, oligomerization, and structure of the proteins comprising the tight junction at the BBB [74,75,76]. These changes may affect paracellular permeability at the BBB independent of changes in expression levels of individual proteins comprising the TJ complex.

In pharmacological studies, BBB disruption is frequently studied as a clinical goal for optimization of drug delivery into the CNS, while its role as an adverse effect of pharmaceutical agents, polypharmacy, and risk factor for brain microenvironment dysregulation is frequently overlooked. APAP is an extraordinarily effective and useful substance used in hundreds of combination medications and consumed by millions of individuals each day in the United States alone [38]. Contrary to APAP popularity, there is limited knowledge on effects of APAP and its potential for drug–drug interactions at the BBB. Previous studies indicate that, while APAP has been shown to preserve brain endothelial cell survival and reduce the neurovascular inflammatory response under oxidative stress in vitro and at low doses in vivo [77,78], APAP at a higher dose (>200 mg/kg) induces cortical oxidative stress and produces reactive astrocytosis without achieving acute liver injury [50,78]. As another example, one prominent site for potential drug interactions at the BBB is P-glycoprotein, a critical efflux transporter at the BBB responsible for restricting entrance of a wide range of substances into the CNS. Our laboratory has previously shown that APAP at 500 mg/kg significantly increases the expression level of P-glycoprotein in brain microvessels and thereby reduces morphine uptake and antinociception [52]. Of particular significance, we demonstrated that APAP activated endothelial signaling pathways such as the constitutive androstane receptor, a nuclear receptor known to be involved in the induction of drug metabolizing enzymes and transporters [52]. In the present study, we further demonstrate that a high dose of APAP elicits drug–drug interactions at the BBB by increasing paracellular permeability and, as a result, can also increase CNS exposure to concomitantly administered drugs that are not transport substrates for P-glycoprotein (i.e., codeine). Importantly, we found that APAP administered at a dose considered safe and effective for analgesia did not alter BBB integrity. Taken together with our previous work, these data imply that an acute, high dose of APAP can have profound effects on cerebral microvascular homeostasis and contribute to unexpected changes in CNS drug disposition.

In addition to drug exposures, the BBB is susceptible to modulation by various physiological stress stimuli. As APAP is the most prevalent and most used analgesic on the market, it is important to note that inflammatory pain by itself could damage tight junction integrity at the BBB. We have previously shown that the induction of inflammatory pain in the hind paw with several different agents (i.e., λ-carrageenan, complete Freund’s adjuvant, formalin) also increases paracellular “leak” of the BBB to sucrose and codeine. Associated with these changes in the λ-carrageenan model are alterations to the tight junction proteins claudin-5, occludin, and ZO-1 as well as the cytoskeletal scaffolding protein, actin [4,5,6]. Studies with the λ-carrageenan model further illustrated prolonged BBB tight junction injury, most prominently at the 3 and 48 h timepoints, which correlated with increased CNS codeine uptake and enhanced codeine analgesia [57]. Given that APAP is commonly used in the presence of pain, it is of our interest to further investigate the effect of APAP in inflammatory pain models and understand their concurrent effects on the function of the BBB.

In conclusion, our novel data show that high-dose APAP increases paracellular permeability of the BBB, an effect that is correlated with increased protein expression of claudin-5 in brain microvessels, an effect that indicates dysregulation of tight junction assembly. The APAP-induced paracellular “leak” contributes to higher CNS exposure to codeine. As noted, many human subjects regularly consume excessive amounts of APAP, including doses greater than or equal to those used in this study on rats, on a daily basis in order to manage acute or chronic pain [43]. Our data suggest that further investigation into effects of APAP on BBB integrity at both the molecular and functional level is warranted. Such studies are likely to yield paradigm-shifting findings that will lead to safer prescribing of APAP and improved formulation of APAP-containing combination products to lower occurrence of accidental overdose of concomitantly administered drugs such as opioids.

## Data Availability

If reasonably requested or needed, data and samples/models will be made available for sharing to qualified parties provided that such a request does not compromise intellectual property interests, interfere with publication, or betray confidentiality. Data that are shared will include standards and notations required to accurately interpret the data, following commonly accepted practices in the field. Data and samples/materials will be available for access and sharing as soon as reasonably possible and no longer than two years after acquisition of the data.

## Supplementary Materials

The following supporting information can be downloaded at: https://www.mdpi.com/article/10.3390/pharmaceutics14050949/s1, Figure S1: Western blot and confocal microscopy demonstrate antibody specificity.

## References

[1] Bernardo-Castro S., Sousa J.A., Bras A., Cecilia C., Rodrigues B., Almendra L., Machado C., Santo G., Silva F., Ferreira L. (2020). Pathophysiology of Blood-Brain Barrier Permeability Throughout the Different Stages of Ischemic Stroke and Its Implication on Hemorrhagic Transformation and Recovery. Front. Neurol..

[2] Abdullahi W., Tripathi D., Ronaldson P.T. (2018). Blood-brain barrier dysfunction in ischemic stroke: Targeting tight junctions and transporters for vascular protection. Am. J. Physiol. Cell Physiol..

[3] Nadareishvili Z., Simpkins A.N., Hitomi E., Reyes D., Leigh R. (2019). Post-Stroke Blood-Brain Barrier Disruption and Poor Functional Outcome in Patients Receiving Thrombolytic Therapy. Cerebrovasc. Dis..

[4] Huber J.D., Witt K.A., Hom S., Egleton R.D., Mark K.S., Davis T.P. (2001). Inflammatory pain alters blood-brain barrier permeability and tight junctional protein expression. Am. J. Physiol. Heart Circ. Physiol..

[5] Huber J.D., Hau V.S., Borg L., Campos C.R., Egleton R.D., Davis T.P. (2002). Blood-brain barrier tight junctions are altered during a 72-h exposure to lambda-carrageenan-induced inflammatory pain. Am. J. Physiol. Heart Circ. Physiol..

[6] Brooks T.A., Hawkins B.T., Huber J.D., Egleton R.D., Davis T.P. (2005). Chronic inflammatory pain leads to increased blood-brain barrier permeability and tight junction protein alterations. Am. J. Physiol. Heart Circ. Physiol..

[7] Cai Z., Qiao P.F., Wan C.Q., Cai M., Zhou N.K., Li Q. (2018). Role of Blood-Brain Barrier in Alzheimer’s Disease. J. Alzheimers Dis..

[8] Ishii M., Iadecola C. (2020). Risk factor for Alzheimer’s disease breaks the blood-brain barrier. Nature.

[9] Sweeney M.D., Sagare A.P., Zlokovic B.V. (2018). Blood-brain barrier breakdown in Alzheimer disease and other neurodegenerative disorders. Nat. Rev. Neurol..

[10] Palmer A.M. (2011). The role of the blood brain barrier in neurodegenerative disorders and their treatment. J. Alzheimers Dis..

[11] Wilson A.C., Clemente L., Liu T., Bowen R.L., Meethal S.V., Atwood C.S. (2008). Reproductive hormones regulate the selective permeability of the blood-brain barrier. Biochim. Biophys. Acta.

[12] Mahringer A., Fricker G. (2010). BCRP at the blood-brain barrier: Genomic regulation by 17beta-estradiol. Mol. Pharm..

[13] Tobwala S., Wang H.-J., Carey J., Banks W., Ercal N. (2014). Effects of Lead and Cadmium on Brain Endothelial Cell Survival, Monolayer Permeability, and Crucial Oxidative Stress Markers in an in Vitro Model of the Blood-Brain Barrier. Toxics.

[14] Calderon-Garciduenas L., Solt A.C., Henriquez-Roldan C., Torres-Jardon R., Nuse B., Herritt L., Villarreal-Calderon R., Osnaya N., Stone I., Garcia R. (2008). Long-term air pollution exposure is associated with neuroinflammation, an altered innate immune response, disruption of the blood-brain barrier, ultrafine particulate deposition, and accumulation of amyloid beta-42 and alpha-synuclein in children and young adults. Toxicol. Pathol..

[15] Oppenheim H.A., Lucero J., Guyot A.C., Herbert L.M., McDonald J.D., Mabondzo A., Lund A.K. (2013). Exposure to vehicle emissions results in altered blood brain barrier permeability and expression of matrix metalloproteinases and tight junction proteins in mice. Part. Fibre Toxicol..

[16] Pimentel E., Sivalingam K., Doke M., Samikkannu T. (2020). Effects of Drugs of Abuse on the Blood-Brain Barrier: A Brief Overview. Front. Neurosci..

[17] Lochhead J.J., Yang J., Ronaldson P.T., Davis T.P. (2020). Structure, Function, and Regulation of the Blood-Brain Barrier Tight Junction in Central Nervous System Disorders. Front. Physiol..

[18] Yousif S., Saubamea B., Cisternino S., Marie-Claire C., Dauchy S., Scherrmann J.M., Decleves X. (2008). Effect of chronic exposure to morphine on the rat blood-brain barrier: Focus on the P-glycoprotein. J. Neurochem..

[19] Yousif S., Chaves C., Potin S., Margaill I., Scherrmann J.M., Decleves X. (2012). Induction of P-glycoprotein and Bcrp at the rat blood-brain barrier following a subchronic morphine treatment is mediated through NMDA/COX-2 activation. J. Neurochem..

[20] Chaves C., Gomez-Zepeda D., Auvity S., Menet M.C., Crete D., Labat L., Remiao F., Cisternino S., Decleves X. (2016). Effect of Subchronic Intravenous Morphine Infusion and Naloxone-Precipitated Morphine Withdrawal on P-gp and Bcrp at the Rat Blood-Brain Barrier. J. Pharm. Sci..

[21] Fiala M., Eshleman A.J., Cashman J., Lin J., Lossinsky A.S., Suarez V., Yang W., Zhang J., Popik W., Singer E. (2005). Cocaine increases human immunodeficiency virus type 1 neuroinvasion through remodeling brain microvascular endothelial cells. J. Neurovirol..

[22] Dhillon N.K., Peng F., Bokhari S., Callen S., Shin S.H., Zhu X., Kim K.J., Buch S.J. (2008). Cocaine-mediated alteration in tight junction protein expression and modulation of CCL2/CCR2 axis across the blood-brain barrier: Implications for HIV-dementia. J. Neuroimmune Pharmacol..

[23] Hawkins B.T., Abbruscato T.J., Egleton R.D., Brown R.C., Huber J.D., Campos C.R., Davis T.P. (2004). Nicotine increases in vivo blood-brain barrier permeability and alters cerebral microvascular tight junction protein distribution. Brain Res..

[24] Manda V.K., Mittapalli R.K., Bohn K.A., Adkins C.E., Lockman P.R. (2010). Nicotine and cotinine increases the brain penetration of saquinavir in rat. J. Neurochem..

[25] Haorah J., Knipe B., Gorantla S., Zheng J., Persidsky Y. (2007). Alcohol-induced blood-brain barrier dysfunction is mediated via inositol 1,4,5-triphosphate receptor (IP3R)-gated intracellular calcium release. J. Neurochem..

[26] Abdul Muneer P.M., Alikunju S., Szlachetka A.M., Haorah J. (2012). The mechanisms of cerebral vascular dysfunction and neuroinflammation by MMP-mediated degradation of VEGFR-2 in alcohol ingestion. Arterioscler. Thromb. Vasc. Biol..

[27] Mahajan S.D., Aalinkeel R., Sykes D.E., Reynolds J.L., Bindukumar B., Adal A., Qi M., Toh J., Xu G., Prasad P.N. (2008). Methamphetamine alters blood brain barrier permeability via the modulation of tight junction expression: Implication for HIV-1 neuropathogenesis in the context of drug abuse. Brain Res..

[28] Ramirez S.H., Potula R., Fan S., Eidem T., Papugani A., Reichenbach N., Dykstra H., Weksler B.B., Romero I.A., Couraud P.O. (2009). Methamphetamine disrupts blood-brain barrier function by induction of oxidative stress in brain endothelial cells. J. Cereb. Blood Flow Metab..

[29] Abdul Muneer P.M., Alikunju S., Szlachetka A.M., Murrin L.C., Haorah J. (2011). Impairment of brain endothelial glucose transporter by methamphetamine causes blood-brain barrier dysfunction. Mol. Neurodegener..

[30] Xue Y., He J.T., Zhang K.K., Chen L.J., Wang Q., Xie X.L. (2019). Methamphetamine reduces expressions of tight junction proteins, rearranges F-actin cytoskeleton and increases the blood brain barrier permeability via the RhoA/ROCK-dependent pathway. Biochem. Biophys. Res. Commun..

[31] Torres E., Gutierrez-Lopez M.D., Mayado A., Rubio A., O’Shea E., Colado M.I. (2011). Changes in interleukin-1 signal modulators induced by 3,4-methylenedioxymethamphetamine (MDMA): Regulation by CB2 receptors and implications for neurotoxicity. J. Neuroinflamm..

[32] Rubio-Araiz A., Perez-Hernandez M., Urrutia A., Porcu F., Borcel E., Gutierrez-Lopez M.D., O’Shea E., Colado M.I. (2014). 3,4-Methylenedioxymethamphetamine (MDMA, ecstasy) disrupts blood-brain barrier integrity through a mechanism involving P2X7 receptors. Int. J. Neuropsychopharmacol..

[33] Weiss N., Miller F., Cazaubon S., Couraud P.O. (2009). The blood-brain barrier in brain homeostasis and neurological diseases. Biochim. Biophys. Acta.

[34] Xiao M., Xiao Z.J., Yang B., Lan Z., Fang F. (2020). Blood-Brain Barrier: More Contributor to Disruption of Central Nervous System Homeostasis Than Victim in Neurological Disorders. Front. Neurosci..

[35] Sun J.J., Xie L., Liu X.D. (2006). Transport of carbamazepine and drug interactions at blood-brain barrier. Acta Pharmacol. Sin..

[36] Wanek T., Romermann K., Mairinger S., Stanek J., Sauberer M., Filip T., Traxl A., Kuntner C., Pahnke J., Bauer F. (2015). Factors Governing P-Glycoprotein-Mediated Drug-Drug Interactions at the Blood-Brain Barrier Measured with Positron Emission Tomography. Mol. Pharm..

[37] Karbownik A., Stanislawiak-Rudowicz J., Stachowiak A., Romanski M., Grzeskowiak E., Szalek E. (2020). The Influence of Paracetamol on the Penetration of Sorafenib and Sorafenib N-Oxide through the Blood-Brain Barrier in Rats. Eur. J. Drug Metab. Pharmacokinet..

[38] Safe Use of Acetaminophen. https://www.chpa.org/about-consumer-healthcare/activities-initiatives/safe-use-acetaminophen.

[39] Kaufman D.W., Kelly J.P., Battista D.R., Malone M.K., Weinstein R.B., Shiffman S. (2019). Five-year trends in acetaminophen use exceeding the recommended daily maximum dose. Br. J. Clin. Pharmacol..

[40] Blieden M., Paramore L.C., Shah D., Ben-Joseph R. (2014). A perspective on the epidemiology of acetaminophen exposure and toxicity in the United States. Expert Rev. Clin. Pharmacol..

[41] Hoban B., Larance B., Gisev N., Nielsen S., Cohen M., Bruno R., Shand F., Lintzeris N., Hall W., Farrell M. (2015). The use of paracetamol (acetaminophen) among a community sample of people with chronic non-cancer pain prescribed opioids. Int. J. Clin. Pract..

[42] Agrawal S., Khazaeni B. (2021). Acetaminophen Toxicity. StatPearls.

[43] Shiffman S., Rohay J.M., Battista D., Kelly J.P., Malone M.K., Weinstein R.B., Kaufman D.W. (2015). Patterns of acetaminophen medication use associated with exceeding the recommended maximum daily dose. Pharmacoepidemiol. Drug Saf..

[44] Larson A.M., Polson J., Fontana R.J., Davern T.J., Lalani E., Hynan L.S., Reisch J.S., Schiodt F.V., Ostapowicz G., Shakil A.O. (2005). Acetaminophen-induced acute liver failure: Results of a United States multicenter, prospective study. Hepatology.

[45] Manchikanti L., Helm S., Fellows B., Janata J.W., Pampati V., Grider J.S., Boswell M.V. (2012). Opioid epidemic in the United States. Pain Physician.

[46] Budnitz D.S., Pollock D.A., Weidenbach K.N., Mendelsohn A.B., Schroeder T.J., Annest J.L. (2006). National surveillance of emergency department visits for outpatient adverse drug events. JAMA.

[47] Drug Enforcement Administration, US Department of Justice (2022). Lists of: Scheduling Actions Controlled Substances Regulated Chemicals.

[48] Prows C.A., Zhang X., Huth M.M., Zhang K., Saldana S.N., Daraiseh N.M., Esslinger H.R., Freeman E., Greinwald J.H., Martin L.J. (2014). Codeine-related adverse drug reactions in children following tonsillectomy: A prospective study. Laryngoscope.

[49] Jones C.M., Mack K.A., Paulozzi L.J. (2013). Pharmaceutical overdose deaths, United States, 2010. JAMA.

[50] Vigo M.B., Perez M.J., De Fino F., Gomez G., Martinez S.A., Bisagno V., Di Carlo M.B., Scazziota A., Manautou J.E., Ghanem C.I. (2019). Acute acetaminophen intoxication induces direct neurotoxicity in rats manifested as astrogliosis and decreased dopaminergic markers in brain areas associated with locomotor regulation. Biochem. Pharmacol..

[51] Ghanem C.I., Perez M.J., Manautou J.E., Mottino A.D. (2016). Acetaminophen from liver to brain: New insights into drug pharmacological action and toxicity. Pharmacol. Res..

[52] Slosky L.M., Thompson B.J., Sanchez-Covarrubias L., Zhang Y., Laracuente M.L., Vanderah T.W., Ronaldson P.T., Davis T.P. (2013). Acetaminophen modulates P-glycoprotein functional expression at the blood-brain barrier by a constitutive androstane receptor-dependent mechanism. Mol. Pharmacol..

[53] Ronaldson P.T., Demarco K.M., Sanchez-Covarrubias L., Solinsky C.M., Davis T.P. (2009). Transforming growth factor-beta signaling alters substrate permeability and tight junction protein expression at the blood-brain barrier during inflammatory pain. J. Cereb. Blood Flow Metab..

[54] Lochhead J.J., McCaffrey G., Sanchez-Covarrubias L., Finch J.D., Demarco K.M., Quigley C.E., Davis T.P., Ronaldson P.T. (2012). Tempol modulates changes in xenobiotic permeability and occludin oligomeric assemblies at the blood-brain barrier during inflammatory pain. Am. J. Physiol. Heart Circ. Physiol..

[55] Takasato Y., Rapoport S.I., Smith Q.R. (1984). An in situ brain perfusion technique to study cerebrovascular transport in the rat. Am. J. Physiol..

[56] Brzica H., Abdullahi W., Reilly B.G., Ronaldson P.T. (2018). A Simple and Reproducible Method to Prepare Membrane Samples from Freshly Isolated Rat Brain Microvessels. J. Vis. Exp..

[57] Hau V.S., Huber J.D., Campos C.R., Davis R.T., Davis T.P. (2004). Effect of lambda-carrageenan-induced inflammatory pain on brain uptake of codeine and antinociception. Brain Res..

[58] Raabe A., Schmitz A.K., Pernhorst K., Grote A., von der Brelie C., Urbach H., Friedman A., Becker A.J., Elger C.E., Niehusmann P. (2012). Cliniconeuropathologic correlations show astroglial albumin storage as a common factor in epileptogenic vascular lesions. Epilepsia.

[59] Salimi H., Klein R.S. (2019). Disruption of the Blood-Brain Barrier during Neuroinflammatory and Neuroinfectious Diseases. Neuroimmune Diseases.

[60] Al-Obaidi M.M.J., Desa M.N.M. (2018). Mechanisms of Blood Brain Barrier Disruption by Different Types of Bacteria, and Bacterial-Host Interactions Facilitate the Bacterial Pathogen Invading the Brain. Cell. Mol. Neurobiol..

[61] Chen Z., Li G. (2021). Immune response and blood-brain barrier dysfunction during viral neuroinvasion. Innate Immun..

[62] Zhang L., Zhou L., Bao L., Liu J., Zhu H., Lv Q., Liu R., Chen W., Tong W., Wei Q. (2021). SARS-CoV-2 crosses the blood-brain barrier accompanied with basement membrane disruption without tight junctions alteration. Signal Transduct. Target. Ther..

[63] Suzuki H., Nishizawa T., Tani K., Yamazaki Y., Tamura A., Ishitani R., Dohmae N., Tsukita S., Nureki O., Fujiyoshi Y. (2014). Crystal structure of a claudin provides insight into the architecture of tight junctions. Science.

[64] Thompson B.J., Sanchez-Covarrubias L., Slosky L.M., Zhang Y., Laracuente M.L., Ronaldson P.T. (2014). Hypoxia/reoxygenation stress signals an increase in organic anion transporting polypeptide 1a4 (Oatp1a4) at the blood-brain barrier: Relevance to CNS drug delivery. J. Cereb. Blood Flow Metab..

[65] Andersson E.A., Mallard C., Ek C.J. (2021). Circulating tight-junction proteins are potential biomarkers for blood-brain barrier function in a model of neonatal hypoxic/ischemic brain injury. Fluids Barriers CNS.

[66] Kazmierski R., Michalak S., Wencel-Warot A., Nowinski W.L. (2012). Serum tight-junction proteins predict hemorrhagic transformation in ischemic stroke patients. Neurology.

[67] Yan B.C., Xu P., Gao M., Wang J., Jiang D., Zhu X., Won M.H., Su P.Q. (2018). Changes in the Blood-Brain Barrier Function Are Associated With Hippocampal Neuron Death in a Kainic Acid Mouse Model of Epilepsy. Front. Neurol..

[68] Schlingmann B., Overgaard C.E., Molina S.A., Lynn K.S., Mitchell L.A., Dorsainvil White S., Mattheyses A.L., Guidot D.M., Capaldo C.T., Koval M. (2016). Regulation of claudin/zonula occludens-1 complexes by hetero-claudin interactions. Nat. Commun..

[69] Coyne C.B., Gambling T.M., Boucher R.C., Carson J.L., Johnson L.G. (2003). Role of claudin interactions in airway tight junctional permeability. Am. J. Physiol. Lung Cell. Mol. Physiol..

[70] Rossa J., Lorenz D., Ringling M., Veshnyakova A., Piontek J. (2012). Overexpression of claudin-5 but not claudin-3 induces formation of trans-interaction-dependent multilamellar bodies. Ann. N. Y. Acad. Sci..

[71] Rossa J., Ploeger C., Vorreiter F., Saleh T., Protze J., Gunzel D., Wolburg H., Krause G., Piontek J. (2014). Claudin-3 and claudin-5 protein folding and assembly into the tight junction are controlled by non-conserved residues in the transmembrane 3 (TM3) and extracellular loop 2 (ECL2) segments. J. Biol. Chem..

[72] Krause G., Protze J., Piontek J. (2015). Assembly and function of claudins: Structure-function relationships based on homology models and crystal structures. Semin. Cell Dev. Biol..

[73] De Mel J.U., Gupta S., Harmon S., Stingaciu L., Roth E.W., Siebenbuerger M., Bleuel M., Schneider G.J. (2021). Acetaminophen Interactions with Phospholipid Vesicles Induced Changes in Morphology and Lipid Dynamics. Langmuir.

[74] Ikenouchi J. (2018). Roles of membrane lipids in the organization of epithelial cells: Old and new problems. Tissue Barriers.

[75] Otani T., Furuse M. (2020). Tight Junction Structure and Function Revisited. Trends Cell Biol..

[76] Vu D.D., Tuchweber B., Raymond P., Yousef I.M. (1992). Tight junction permeability and liver plasma membrane fluidity in lithocholate-induced cholestasis. Exp. Mol. Pathol..

[77] Tripathy D., Grammas P. (2009). Acetaminophen protects brain endothelial cells against oxidative stress. Microvasc. Res..

[78] Naziroglu M., Uguz A.C., Kocak A., Bal R. (2009). Acetaminophen at different doses protects brain microsomal Ca^2+^-ATPase and the antioxidant redox system in rats. J. Membr. Biol..

