# Supplementary material for: High-Dose Acetaminophen Alters the Integrity of the Blood–Brain Barrier and Leads to Increased CNS Uptake of Codeine in Rats
Source: Pharmaceutics. 2022 Apr 27;14(5):949. doi: 10.3390/pharmaceutics14050949 (PMC9144323; doi:10.3390/pharmaceutics14050949)
Supplement: Supplementary file 1 [file pharmaceutics-14-00949-s001.zip › pharmaceutics-1665452-supplementary.pdf]

pharmaceutics

Supplementary Materials: High-Dose Acetaminophen Alters the Integrity of the Blood–Brain Barrier and Leads to Increased CNS Uptake of Codeine in Rats

Junzhi Yang, Robert D. Betterton, Erica I. Williams, Joshua A. Stanton, Elizabeth S. Reddell, Chidinma E. Ogbonnaya, Emma Dorn, Thomas P. Davis, Jeffrey J. Lochhead and Patrick T. Ronaldson

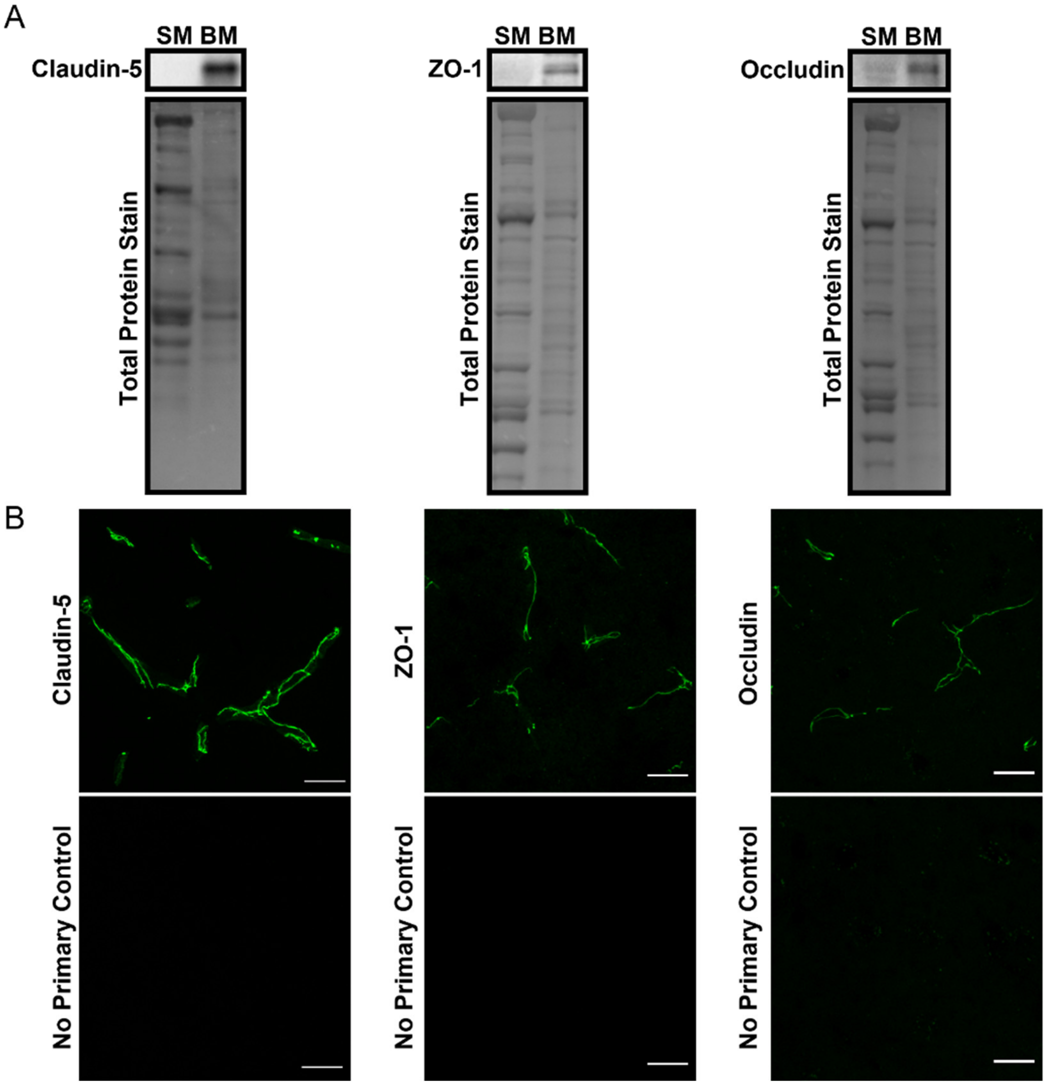

Figure S1. Western blot and confocal microscopy demonstrate antibody specificity. (**A**) Western blot (top) with claudin-5 antibody (Cat #40-6100; Thermo Fisher Scientific) shows undetectable level of claudin-5 in rat skeletal muscle (SM) compared to rat brain microvessel (BM). Antibody specificity is further demonstrated in confocal microscopy images (bottom) in which no signal is detected in isolated rat brain microvessel without the primary antibody. Western blot (top) with ZO-1 antibody ZO-1 (Cat #40-2200; Abcam, Inc.) detects little to no ZO-1 in rat skeletal muscle compared to rat brain microvessel. Antibody specificity is further demonstrated in confocal microscopy images (bottom) in which no signal is detected in isolated rat brain microvessel without primary antibody. Western blot (top) with occludin antibody (Cat #40-6100; Thermo Fisher Scientific) detects low level of occludin in rat skeletal muscle in comparison with rat brain microvessel. (**B**) Antibody specificity is further demonstrated in confocal microscopy images (bottom) in which no signal is detected in isolated rat brain microvessel without primary antibody.
